# Supplementary material for: Structural and Functional Insights into the Stealth Protein CpsY of Mycobacterium tuberculosis
Source: Biomolecules. 2023 Nov 3;13(11):1611. doi: 10.3390/biom13111611 (PMC10668966; doi:10.3390/biom13111611)
Supplement: Supplementary file 1 [file biomolecules-13-01611-s001.zip › biomolecules-2692156-supplementary.pdf]

Supplementary Material

Structural and Functional Insights into the Stealth Protein CpsY of *Mycobacterium tuberculosis*

Dafeng Liu [1], Cai Yuan [2], Chenyun Guo [1], Mingdong Huang [3,*] and Donghai Lin [1,*]

[1] MOE Key Laboratory of Spectrochemical Analysis & Instrumentation, Key Laboratory of Chemical Biology of Fujian Province, College of Chemistry and Chemical Engineering, Xiamen University, Xiamen 361005, China
[2] College of Biological Science and Engineering, Fuzhou University, Fuzhou 350108, China
[3] College of Chemistry, Fuzhou University, Fuzhou 350108, China
* Correspondence: hmd_lab@fzu.edu.cn (M.H.); dhlin@xmu.edu.cn (D.L.)

Table S1. Search for structural homologs using DALI

Rank	PDB code	Z-score	RMSD (Å)	LALI	NRES	Identity (%)	Description
1	7SJ2	29.6	2.0	260	459	33	N-acetylglucosamine-1-phosphotransferase subunits
2	5GVV	11.2	3.9	187	392	9	glycosyl transferase family 8
3	6U4B	11.1	3.7	204	570	7	wbbm protein
4	3TZT	10.9	3.7	182	234	10	glycosyl transferase family 8
5	7UI7	10.6	3.8	190	613	9	xylosyl- and glucuronyl transferase large1

Table S2. Kinetic parameters of ΔS2 and ΔS3

Construct	UDP-GlcNAc		Glucan	
	K_m (μM)	K_{cat} (min^{-1})	K_m (μM)	K_{cat} (min^{-1})
$CpsY^{201\text{-}520}$	26.3±1.9	11.2±0.3	463.6±11.8	67.5±2.7
ΔS2	10.3±0.8	29.3±0.7	187.2±3.7	100.4±1.5
ΔS3	16.9±0.5	19.7±0.4	267.8±2.8	85.6±1.7

Note: Kinetic parameters were determined using continuous phosphate detection with increasing UDP-GlcNAc concentrations or increasing glucan concentrations.

Figure S1

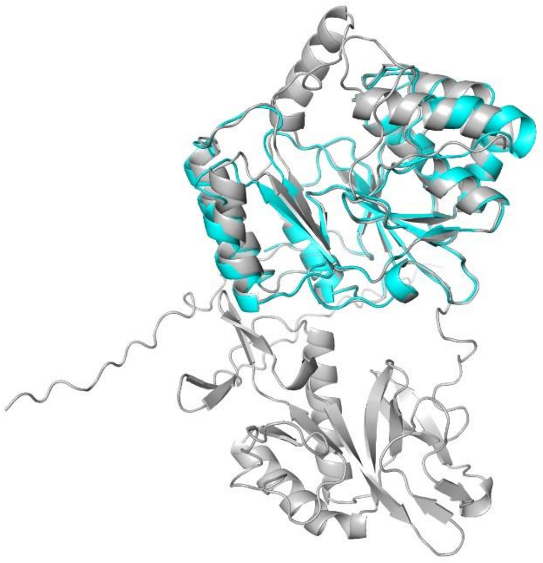

Figure S1. Structural comparison between the crystal structure of CpsY$^{201-520}$ (in cyan) and the full-length CpsY model predicted by AlphaFold2 (in gray) [1, 2]**.** The structural comparison reveals that the overall folds of the two are similar. However, there is a large root mean square deviation (RMSD) value of 1.2 Å for all atoms.

Figure S2

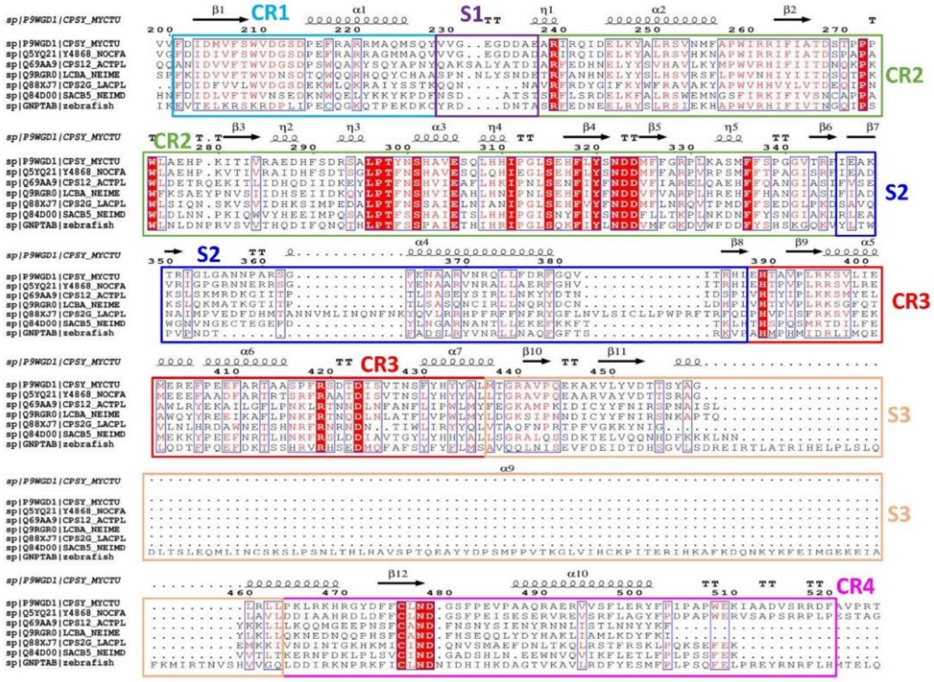

Figure S2. Sequence alignments of stealth proteins. The alignment utilizes the ClustalW default color scheme, with greater color intensity denoting conservation among amino acids, as opposed to non-conserved residues. The alignment incorporates the following stealth proteins: P9WGD1, corresponding to CpsY from *Mycobacterium tuberculosis*; Q5YQ21, representing NFA_48680 from *Nocardia farcinica*; Q69AA9, signifying Cps12A from *Actinobacillus pleuropneumoniae*; Q9RGR0, indicating LcbA from *Neisseria meningitidis*; Q88XJ7, representing Cps2G from *Lactiplantibacillus plantarum*; Q84D00, denoting SacB from *Neisseria meningitidis serogroup A*; GNPTAB, from Zebrafish. Four conserved regions (CR1-CR4) and three spacer segments (S1-S3) are colored as represented in Figure 1A.

Figure S3

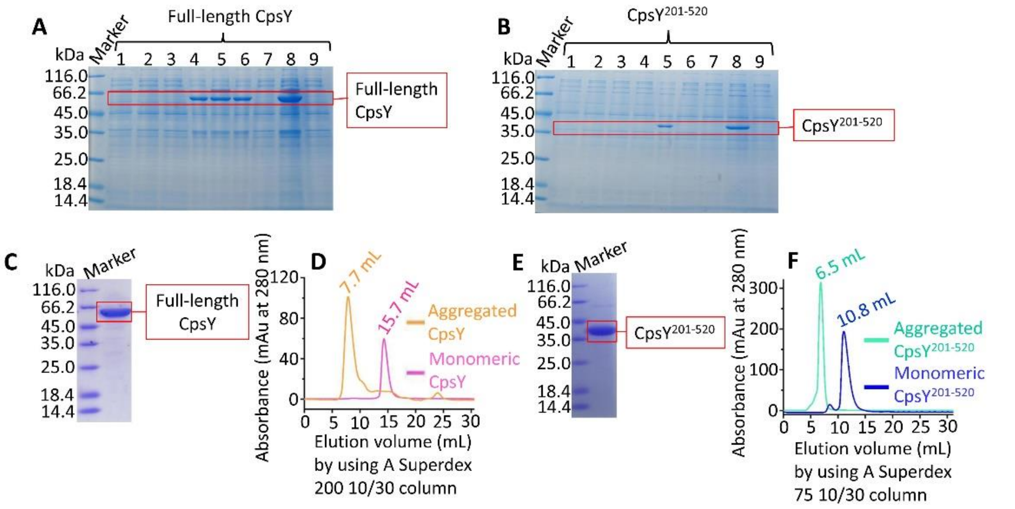

Figure S3. Expression and purification of different recombinant CpsY proteins. (A-B) SDS-PAGE analysis of small-scale expression of CpsY and CpsY$^{201-520}$ in different expression conditions (different induction temperature, time and IPTG concentrations). For small-scale expression of CpsY. Lane 1, 2 and 3: 0.1 mM IPTG; Lane 4, 5 and 6: 0.5 mM IPTG; Lane 7, 8 and 9: 1.0 mM; Lane 1, 4 and 7: 16°C for 18 h; Lane 2, 5 and 8: 25°C for 12 h; Lane 3, 6 and 9: 37°C for 4 h. For small-scale expression of CpsY$^{201-520}$. Lane 1, 2 and 3: 0.1 mM IPTG; Lane 4, 5 and 6: 0.5 mM IPTG; Lane 7, 8 and 9: 1.0 mM; Lane 1, 4 and 7: 16°C for 18 h; Lane 2, 5 and 8: 25°C for 12 h; Lane 3, 6 and 9: 37°C for 4 h. Results showed that CpsY and CpsY$^{201-520}$ were expressed using the optimal expression condition (25°C, 1 mM IPTG and 12 h) (C) The result of SDS-PAGE (12%) analysis showed that CpsY possessed high purity. (D) The elution peaks of aggregated and monomeric CpsY were determined using a Superdex 200 10/30 column: 7.7 mL in buffer I (500 mM NaCl, 20 mM Tris-HCl, pH 7.4) for CpsY aggregate, and 15.7 mL in buffer II (20 mM Tris-HCl, pH 7.4, 500 mM NaCl, 5 mM DTT and 5% glycerol) for monomeric CpsY. (E) The result of SDS-PAGE (12%) analysis showed high purity for CpsY$^{201-520}$. (F) Elution peak of aggregated CpsY$^{201-520}$ was 6.5 mL in buffer I (500 mM NaCl, 20 mM Tris-HCl, pH7.4) using a superdex 75 10/30 column, elution peak at 10.8 mL for monomeric CpsY$^{201-520}$ in buffer II (20 mM Tris-HCl, pH7.4, 500 mM NaCl, 5 mM DTT and 5% glycerol).

Figure S4

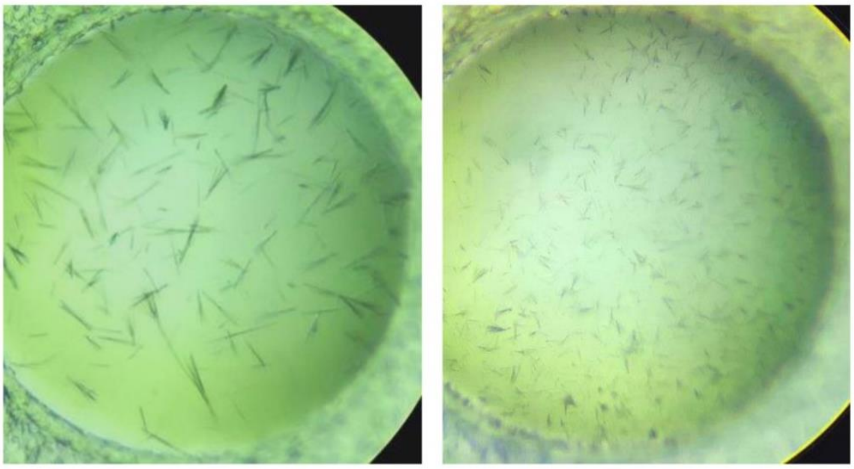

Figure S4. Protein crystals of full-length CpsY were achieved, however, attempts to refine them into a diffracting state failed.

Figure S5

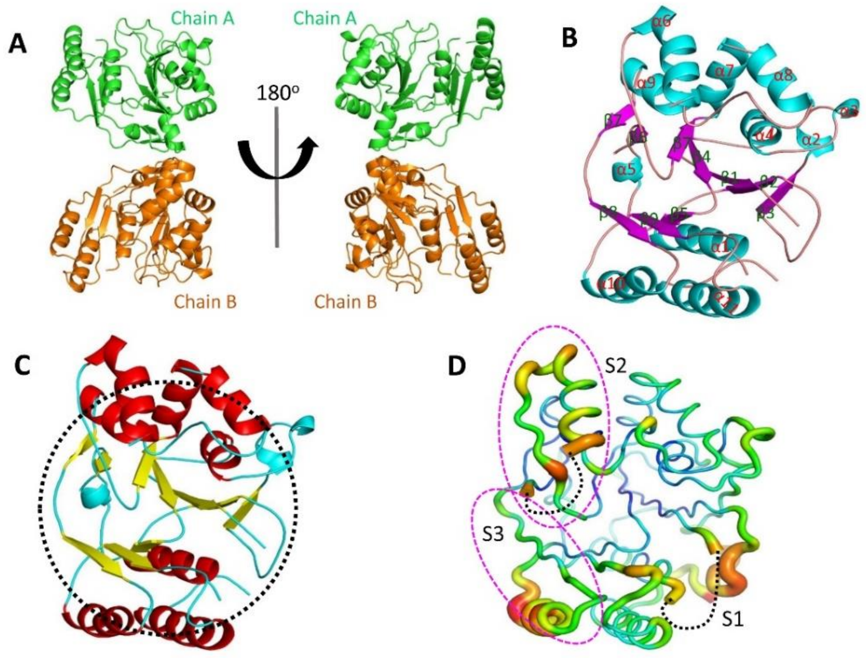

Figure S5. Gaining insight into the crystal structure of CpsY$^{201\text{-}520}$. (A) A schematic diagram of the crystal structure showcases two molecules within a single asymmetric unit, depicted through an 180° rotation. Chain A is visualized in green, whereas chain B is displayed in orange. (B) Depiction of the crystal structure of chain B, with helices, sheets, and loops colored in cyan, magenta and orange, respectively. (C) The interior of CpsY$^{201\text{-}520}$ reveals the presence of three nearly parallel β-sheets composed of five, three, and two strands, distinguished by a yellow hue. Adjacent to these β-sheets are three α-helices on one side and five on the other, rendered in red. Noteworthy is the depiction of a deep cavity, demarcated by a black dashed oval. (D) The structural flexibility of CpsY$^{201\text{-}520}$ is reflected through B-factor values. Utilizing the default PyMOL color scheme, amino acids with higher B-factor values are shaded redder, signifying increased structural flexibility compared to amino acids with lower B-factor values. Spacer segments (S1, S2 and S3) stand out due to both elevated B-factor values and notable flexibility. Additionally, dashed lines in the structure of CpsY$^{201\text{-}520}$ represent a loop with invisible electron densities.

Figure S6

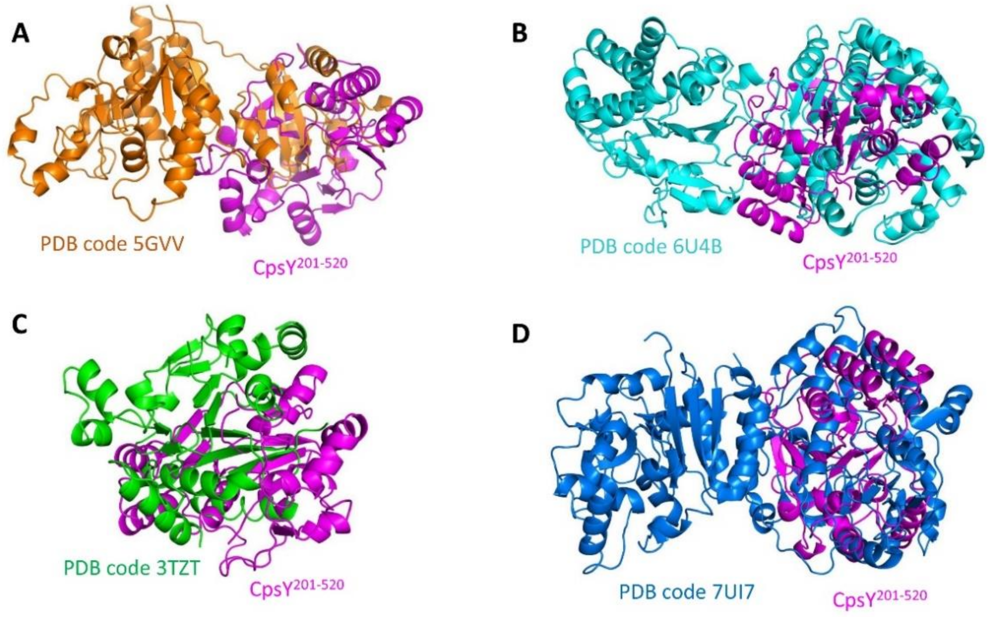

Figure S6. Structural comparison between CpsY$^{201\text{-}520}$ and other proteins. CpsY$^{201\text{-}520}$ is depicted in magenta. The comparisons encompass the following proteins: (A) A protein glycosyl transferase family 8 (PDB code 5GVV) in *Streptococcus pneumoniae* TIGR4, depicted in orange. (B) The wbbm protein (PDB code 6U4B) from *Klebsiella pneumoniae*, rendered in cyan. (C) A glycosyl transferase family 8 protein (PDB code 3TZT) from *Anaerococcus prevotii*, rendered in green. (D) The xylosyl- and glucuronyltransferase large1 protein (PDB code 7UI7) in *Homo sapiens*, showcased in marine.

Figure S7

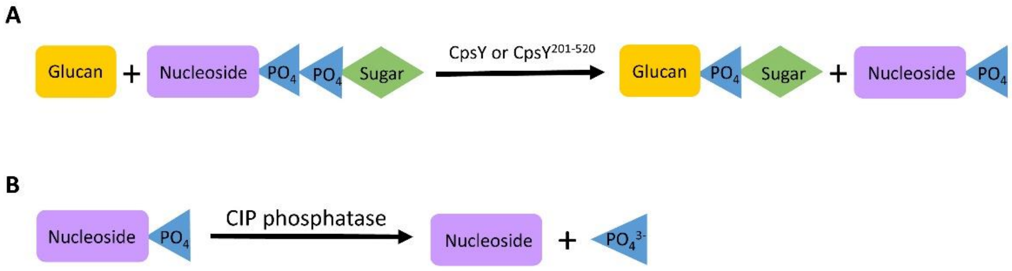

Figure S7. Exploration of phosphate ion generation. (A) Either CpsY or $CpsY^{201-520}$ was used to catalyze the phosphotransfer reaction. (B) The production of phosphate ions in the final outcome was measured to assess the activities of CpsY and $CpsY^{201-520}$.

Figure S8

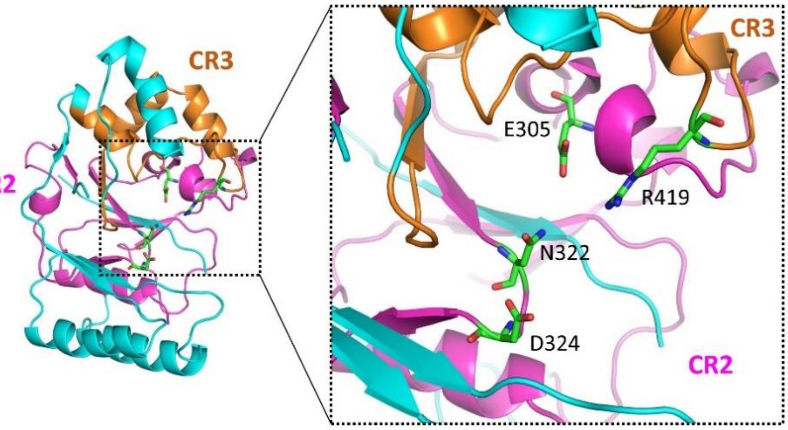

Figure S8. Several key residues present in the conserved regions 2 and 3 (CR2 and CR3) of CpsY$^{201-520}$ (in cyan). Residues E305, N322 and D324 are located in the CR2 region (in magenta), while R419 is positioned in the CR3 region (in orange).

Figure S9

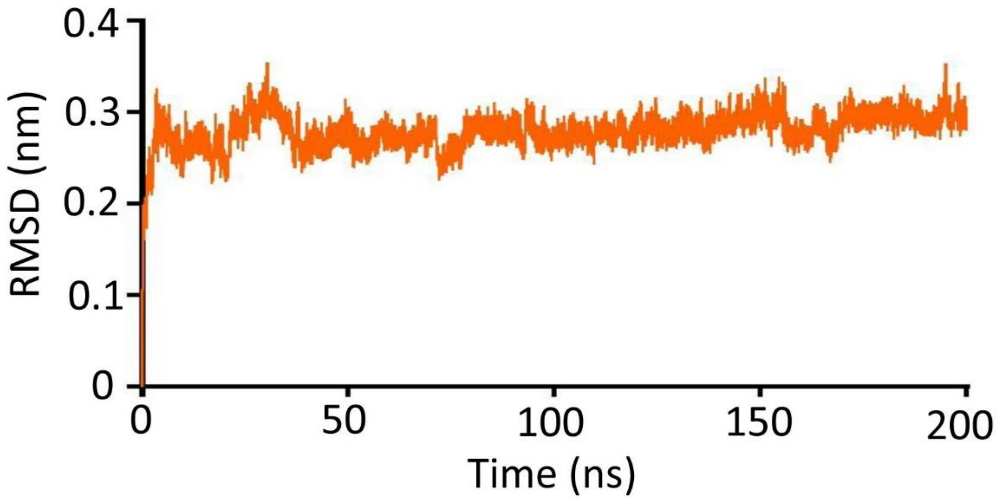

Figure S9. Root mean square deviation (RMSD) in CpsY$^{201-520}$ backbone atoms. We have analyzed RMSD of backbone atoms of all the systems for an overall time of 200 ns simulation run, and found that all the systems have converged within a range of 50-200 ns. RMSD was used to measure the deviation of the coordinates of a particular atom with respect to a reference structure and assess whether the simulation system has reached stability. A stable RMSD indicates that the corresponding atom possesses stability.

Figure S10

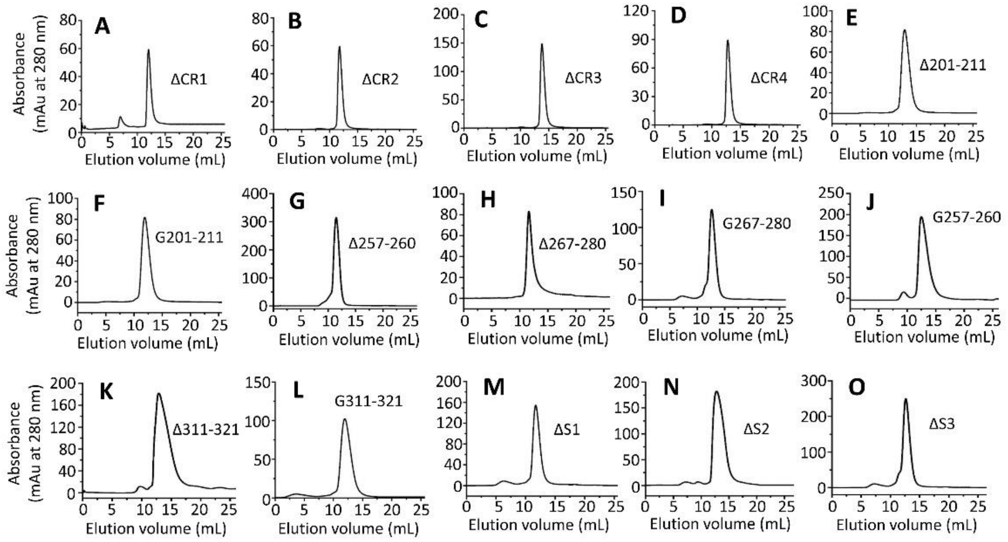

Figure S10. Size-exclusion chromatography (SEC) of purified CpsY$^{201-520}$ variants. Protein purification was performed by using a Superdex 75 10/30 column and buffer II (20 mM Tris-HCl, pH7.4, 500 mM NaCl, 5 mM DTT and 5% glycerol). Each recombinant protein exhibited a distinguishable single peak, indicating a consistent molecular size distribution within the eluted fractions.

References

1. Millán, C.; Keegan, R. M.; Pereira, J.; Sammito, M. D.; Simpkin, A. J.; McCoy, A. J.; Lupas, A. N.; Hartmann, M. D.; Rigden, D. J.; Read, R. J., Protein-structure prediction revolutionized. *Nature* **2021,** 596, (26), 2.
2. Jumper, J.; Evans, R.; Pritzel, A.; Green, T.; Figurnov, M.; Ronneberger, O.; Tunyasuvunakool, K.; Bates, R.; Žídek, A.; Potapenko, A.; Bridgland, A.; Meyer, C.; Kohl, S. A. A.; Ballard, A. J.; Cowie, A.; Romera-Paredes, B.; Nikolov, S.; Jain, R.; Adler, J.; Back, T.; Petersen, S.; Reiman, D.; Clancy, E.; Zielinski, M.; Steinegger, M.; Pacholska, M.; Berghammer, T.; Bodenstein, S.; Silver, D.; Vinyals, O.; Senior, A. W.; Kavukcuoglu, K.; Kohli, P.; Hassabis, D., Highly accurate protein structure prediction with AlphaFold. *Nature* **2021,** 596, (7873), 583-589.
